# A Rapid Lysostaphin Production Approach and a Convenient Novel Lysostaphin Loaded Nano-emulgel; As a Sustainable Low-Cost Methicillin-Resistant *Staphylococcus aureus* Combating Platform

**DOI:** 10.3390/biom10030435

**Published:** 2020-03-12

**Authors:** Hanzada T. Nour El-Din, Noha M. Elhosseiny, Mohamed A. El-Gendy, Azza A. Mahmoud, Manal M. M. Hussein, Ahmed S. Attia

**Affiliations:** 1 Department of Microbiology and Immunology, Faculty of Pharmacy, Cairo University, Cairo 11562, Egypt; Hanzada.noreldin@pharma.cu.edu.eg (H.T.N.E.-D.); noha.elhuseiny@pharma.cu.edu.eg (N.M.E.);; 2 Department of Pharmaceutics and Pharmaceutical Technology, Faculty of Pharmaceutical Sciences and Pharmaceutical Industries, Future University in Egypt, Cairo 11835, Egypt; Mohamed.Emad@fue.edu.eg (M.A.E.-G.); azza.ahmed@fue.edu.eg (A.A.M.); 3 Department of Microbiology & Immunology, School of Pharmacy, New Giza University, Giza 12256, Egypt

**Keywords:** lysostaphin, sustainable, factorial-design, optimization, low-cost, nano-emulgel, skin infections

## Abstract

*Staphylococcus aureus* is a Gram-positive pathogen that is capable of infecting almost every organ in the human body. Alarmingly, the rapid emergence of methicillin-resistant *S.* *aureus* strains (MRSA) jeopardizes the available treatment options. Herein, we propose sustainable, low-cost production of recombinant lysostaphin (rLST), which is a native bacteriocin destroying the staphylococcal cell wall through its endopeptidase activity. We combined the use of *E. coli* BL21(DE3)/pET15b, factorial design, and simple Ni-NTA affinity chromatography to optimize rLST production. The enzyme yield was up to 50 mg/L culture, surpassing reported systems. Our rLST demonstrated superlative biofilm combating ability by inhibiting staphylococcal biofilms formation and detachment of already formed biofilms, compared to vancomycin and linezolid. Furthermore, we aimed at developing a novel rLST topical formula targeting staphylococcal skin infections. The phase inversion composition (PIC) method fulfilled this aim with its simple preparatory steps and affordable components. LST nano-emulgel (LNEG) was able to extend active LST release up to 8 h and cure skin infections in a murine skin model. We are introducing a rapid, convenient rLST production platform with an outcome of pure, active rLST incorporated into an effective LNEG formula with scaling-up potential to satisfy the needs of both research and therapeutic purposes.

## 1. Introduction

Since their clinical introduction in the 1930s, antibiotics have highly impacted human morbidity and mortality. Unfortunately, decades of antibiotics mis-use has applied selective pressure on pathogens, which results in unprecedented antimicrobial resistance [1,2]. *S. aureus* is a leading cause of hospital and community infections worldwide [3]. However, the most recent antimicrobial for combating staphylococcal infections was discovered 30 years ago. To fill this gap in a way that considerably lower the resistance potential, new non-conventional ways had to be explored. Investigated alternatives include plant-derived compounds [4], bacteriophages and phage lysins [5], RNA-based therapeutics [6], antimicrobial adjuvants [7], and antimicrobial peptides (AMP) [8].

Bacteriocins, which are one subgroup of AMPs, are ribosomal-synthesized proteinaceous bactericidal compounds produced by immune bacteria including one of which is lysostaphin (LST). LST, which is also termed as a bacteriolysin, is an endopeptidase discovered in the 1960s as an anti-staphylococcal agent destroying the bacterial cell wall, which makes it a promising therapeutic alternative to treat staphylococcal infections, starting from skin and moving to systemic infections [9,10]. The pharmaceutical industry has been revolutionized by a variety of formulas, among which are the nano-emulsions (NEs). NE is defined as a disperse system with droplets of less than 100 nm [11]. This small size not only imparts advantageous physical properties, such as optical clarity and elastic behavior, but also makes NEs stable against sedimentation and creaming [12]. Moreover, NEs are thermodynamically stable, and can be used when aiming at controlled drug release. Topically, NEs, specifically the water in oil ones, has better adhesion on the skin surface, which leads to a larger concentration gradient and, hence, better targeting for skin infections. In addition, topical NEs are non-greasy, easily spreadable, and removable when enhancing patients’ compliance [13]. Commercially, NEs are gaining more focus due to their simple preparatory procedures and the affordability of the materials in use. This reduces their overall production cost [14] and, as a result, they are already used as vehicles for drugs active against herpes labialis, topical fungal, and bacterial infections and vaginitis [15].

In the present study, we aim to optimize recombinant lysostaphin (rLST) production and treat staphylococcal skin infections topically under the umbrella of swiftness, affordability, and simplicity. This could enable countries with minimal resources to make full use of lysostaphin applications with the potential for scale up production.

## 2. Materials and Methods

### 2.1. Statement of Ethical Approval

All animal procedures were approved by the Research Ethics Committee of the Faculty of Pharmacy, Cairo University (approval# MI 1613), following the Guide for the Care and Use of Laboratory Animals published by the Institute of Laboratory Animal Research (Washington, DC, USA).

### 2.2. Bacterial Strains and Culture Conditions

Staphylococcal strains used in this study included: *S. aureus* strains Newman [16] and USA300, which is a multiple antibiotic resistant and community-acquired strain [17], the *S. simulans* strain TNK3 [18], the *S. epidermidis* strain ATCC 12228 (ATCC), and the *S. lugdunensis* strain N920143 [19]. *Escherichia coli* strain TOP10 (Invitrogen) was used for cloning and strain BL21 (DE3) [20] used for the rLST production. Staphylococcal strains were grown aerobically at 37 °C in tryptic soy broth (TSB). For *E. coli* strains, they were grown aerobically at 37 °C in Luria Bertani (LB) broth. When appropriate, LB was supplemented with ampicillin at a final concentration of 100 µg/mL.

### 2.3. Cloning and Expression of Recombinant Lysostaphin in E. coli

Primer pair AA662 (5′-GGGcatatgGCTGCAACACATGAACA-3′, NdeI site underlined) and AA663 (5′-GGGctcgagTTACTTTATAGTTCCCCAAAG-3′, XhoI site underlined and stop codon in italics) was used to amplify a 738 bp fragment encoding mature lysostaphin [21], using purified chromosomal S. simulans DNA as a template. The PCR product was column purified, and then double digested using NdeI and XhoI (NEB). The digested PCR product was then ligated into plasmid pET15b (Novagen, Madison, MA, USA) and transformed into the E. coli TOP10 cells. The resulting plasmid was verified by DNA sequencing and designated pET15b-lyso. Then it was transformed into E. coli BL21 (DE3) for protein production. To screen for successful lysostaphin production, a BL21/ pET15b-lyso overnight culture was used to inoculate fresh LB medium in a ratio of 1:100, and grown at 37 °C to mid-log phase, which is followed by induction with 0.5 mM isopropyl-D-1-thiogalactopyranoside (IPTG) for 3 h at 30 °C. The cells were collected by centrifugation at 4000× *g* at 4 °C, and disrupted by sonication at 40% amplitude, and 3-s pulses for 10 min. The lysate was centrifuged and the clear supernatant was purified with Ni-NTA resin (Qiagen, Hilden, Germany) as described earlier [22] and, then, analyzed on a 12.5% sodium dodecyl sulphate polyacrylamide gel electrophoresis (SDS-PAGE). When needed, the recombinant lysostaphin (rLST) elution buffer was exchanged with 0.1 M phosphate buffer pH 7.5 using the Zeba™ Spin Desalting Columns, 7K MWCO (Thermo Fisher, Waltham, WA, USA).

### 2.4. Optimization of Recombinant Lysostaphin Production

Full factorial design using the statistical software package Minitab 16 (Minitab Inc., State College, Pennsylvania, PA, USA) was applied to evaluate the influence of five independent factors (temperature, IPTG-concentration, growth medium type, induction time, and agitation speed) against the dependent variable of the rLST yield in mg/L culture. The low (−), basal (0), and high (+) levels for the studied factors were as follows, respectively: temperature (16, 30, and 37 °C), IPTG concentration (0.25, 0.5, and 1 mM), type of growth medium (nutrient broth (NB), LB, and TSB), induction time (3, 6, and 18 h), and agitation speed (100, 180, and 250 rpm). Following each run, the purified lysostaphin was quantitated using a nanophotometeric device (Implen, California, CA, USA) and the QuantiPro BCA assay kit (Sigma-Aldrich, St. Louis, Missouri, MI, USA). In addition, aliquots of each elution fraction were analyzed by SDS-PAGE.

### 2.5. Analyses of the Different Factors and Their Interactions on the Production of rLST

The standardized effect of each factor (E-value) was calculated using Minitab 16. The E-value magnitude of the tested factor indicated its effect or its significance in affecting the response, while its sign indicated its positive or negative influence on the responses. The interacting factors were determined by presenting the analysis of variance (ANOVA). The main and interaction effects of each factor having *p* values ≤ 0.05 were considered as potentially significant.

To investigate the interplay of the evaluated factors, interaction plots, expressed in terms of rLST conc. mg/ L, were constructed. Parallel lines indicate the absence of an interaction, whereas non-parallel lines indicate the presence of an interaction. The F-value of this test determines whether a group of terms is associated with the response. A sufficiently large F-value indicates statistical significance.

### 2.6. Lysostaphin Activity Assay

The bacteriolytic activity of rLST was determined as described before [23]. Overnight cultures of the staphylococcal strains mentioned above (18 h) were pelleted and the cells were then resuspended in a 0.1 M phosphate buffer pH 7.5 to OD_600_ of 0.25. The cell suspension was preincubated at 37 °C for 10 min, and then 50 µL of rLST preparation was added. One unit (U) of activity is defined as the amount of preparation causing 50% reduction in the turbidity of a 6-mL cell suspension within 10 min at 37 °C in a 10-mm cuvette.

### 2.7. Biofilms Combating Assays

#### 2.7.1. Determination of the Minimum Inhibitory Concentration (MIC)

MIC of rLST, vancomycin, and linezolid were determined for all the five tested staphylococcal strains using the two-fold dilution method according to the Clinical and Laboratory Standards Institute (CLSI) guidelines [24].

#### 2.7.2. Biofilm Formation Inhibition Assay (Pre-Exposure)

Staphylococcal cells were gown for 18 h in TSB and then cultures were normalized to OD_600_ of 1 and diluted 1:100 in fresh TSB. Diluted cultures (100 µL) were mixed with equal volume of either rLST (20 U), or a solution containing the MIC value of vancomycin or linezolid to have a final concentration of half the MIC (for the respective strain) and placed in the wells of an untreated polystyrene, 96-well flat-bottomed plate. The plates were incubated statically at 37 °C for 24 h. Wells were washed three times with phosphate buffered saline (PBS). The washing step was done by gently submerging the plate in a small tub of PBS, and then shaking the water off and, lastly, blot dry on a stack of paper towels and leave to dry overnight [25]. Adherent cells were stained with crystal violet (0.4% *w*/*v*) at room temperature for 15 min. Then wells were washed, 150 µL of absolute ethanol were added, and absorbance at OD_595_ was recorded. The biofilm formation ability was evaluated using a biofilm formation index [BFI]: (ODCV Biofilm - ODCV Control)/ODPlanktonic [26,27].

#### 2.7.3. Biofilm Detachment Assay (Post-Exposure)

An 18 h TSB cultures of staphylococci were normalized to OD_600_ of 1, and then diluted 1:100 with fresh TSB. Individual wells of a 96-well flat-bottomed plates were filled with 0.1 mL aliquots of the diluted culture. The plates were incubated for 24 h at 37 °C. Then the OD_600_ of the wells was measured after the incubation for normalization purposes. Growth medium and the unattached cells were discarded, and the biofilms were washed three times with PBS. A washing step was done by gently submerging the plate in a small tub of PBS, then shaking the buffer off, and, lastly, blot dry the plate on a stack of paper towels and leave to dry overnight. Next, 50 µL of either rLST (20 U), vancomycin (400 µg/mL), or linezolid (800 µg/mL) were mixed with 50 µL of TSB/well and added to the plates. Untreated biofilms (100% reference values) were obtained by adding 50 µL of ultrapure water instead of the antimicrobial agents. The plates were incubated at 37 °C for 24 h [28]. The rest of the protocol was done as described above.

### 2.8. Incorporation of the rLST into Nano-Emulgel Preparation

The nano-emulgel was prepared using the phase inversion composition method (PIC) [29]. The vehicle was prepared by mixing 0.2 g isopropyl myristate as the oil phase, 0.7 g Tween 80 as a surfactant, and 0.1 g polyethylene glycol 400 as a co-surfactant by vortexing for 60 s. Then, a 0.67 g aqueous phase containing 1 mg rLST were added stepwise with continuous mixing.

#### 2.8.1. Characterization of the rLST Nano-Emulgel

Particle size and morphological examination of the prepared formula were done using a high resolution-transmission electron microscope (HR-TEM - JEOL2100, JEOL Ltd., Akishima, Japan) coupled with a Gatan axis-mount 2kx2k digital camera (Gatan, Inc., California, CA, USA).

#### 2.8.2. In-Vitro Release Study

The experiment was conducted in a vertical Franz diffusion cell apparatus with an acceptor compartment volume of 6.8 mL, which has a membrane filter (HT-450 Tuffryn membrane with 0.45 µm pore size, Pall Life Science, New York, NY, USA) and diffusion area of 1.767 cm^2^ (MicroettePlus™, Hanson Research, California, CA, USA). For the donor compartment, an amount of 0.25–0.3 g of the formulation was initially set on the cellulose membrane and the appearance of the lysostaphin protein was monitored in the receptor compartment, containing PBS, pH 7.4) at 0, 0.5, 1, 2, 3, 4, 5, 6, 7, 8, 12, and 24 h. The withdrawn samples were then measured for the rLST protein content and the activity of the released rLST was determined as described above.

### 2.9. Testing the Efficacy of the rLST Nano-Emulgel in an In-Vivo Murine Skin Model of Infection

An in vivo murine methicillin-resistant *S. aureus* strains (MRSA) skin infection study was conducted as described before [30,31]. One day prior to the experiment, the backs of the six-eight-week-old BALB/C mice (Theodor Bilhariz Research Institute, Giza, Egypt) were shaved. Three mice groups (n = 5) received an intradermal injection (40 µL) of MRSA USA300 containing 3 × 10^7^ CFU. Forty-eight hours after infection, an open wound/abscess was formed at the site of injection. The first group was treated topically with the rLST nano-emulgel. The rLST nano-emulgel was prepared using the PIC method and it was applied three times daily using a disposable sterile cotton swab for each mouse at every application event. The second group was treated with the vehicle alone, while the control group did not receive any treatment. The respective mice were treated three times daily for five days. Twenty-four hours after the last treatment, mice were humanely euthanized using an over-dose of anesthesia followed by cervical dislocation. The wound area (~1 cm^2^) was excised and homogenized in 0.5 mL of PBS. The homogenized skins were serially diluted and, then, aliquots of each dilution were plated on Mannitol Salt Agar (MSA) plates for viable counting. The extent of the lesion formation and ulceration was recorded using a digital camera.

## 3. Results

### 3.1. Cloning and Recombinant Lysostaphin Production

**Figure 1:**
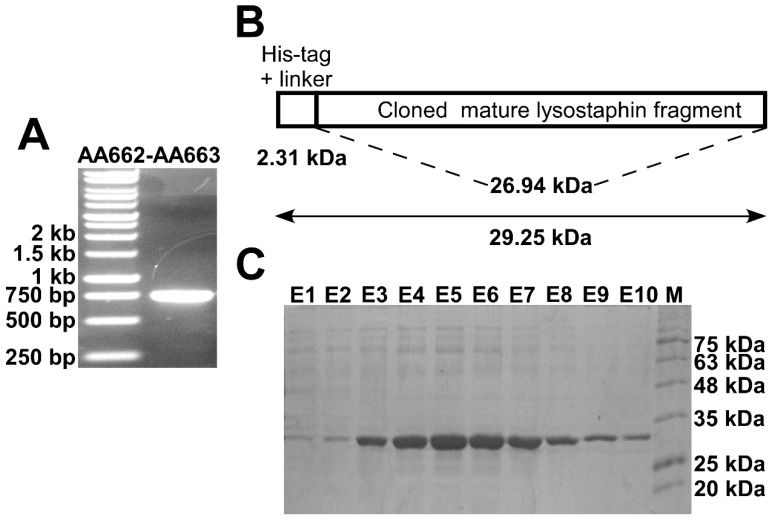
**Cloning, production, and purification of rLST in *E. coli*.** (**A**). Photograph of a 1% agarose gel showing the PCR product of ~740 bp produced by the reaction using the primer pair AA662-AA663. The first lane of the gel contained a DNA ladder for size estimation. (**B**). Schematic diagram showing the expected size of the encoded lysostaphin His-tagged protein fragment based on the ProtParam tool. (**C**). Photograph of a 12.5% SDS-PAGE gel stained with Coomassie Brilliant Blue showing elution fractions (E1–10) following Ni-NTA agarose beads purification. A prominent band representing the purified protein appeared ~29 kDa. The last lane contained a protein molecular weight marker for the size estimation.

The DNA fragment encoding the mature lysostaphin estimated to be 738 bp was successfully amplified (Figure 1A). Then it was cloned in the expression vector pET15b and expressed. The His-tagged fragment was predicted to be approximately 29 kDa (Figure 1B) based on the prediction using the ProtParam tool (https://web.expasy.org/protparam/) [32]. A band was visualized on an SDS-PAGE gel corresponding to the predicted size of the produced fragment (Figure 1C).

### 3.2. Optimization of rLST Production

The main effects analyses indicated that changing the growth media from NB to the much richer medium TSB had the greatest positive effect (E-value = 13.362, *p* < 0.001). On the other hand, increasing the IPTG concentration from 0.25 to 1 mM was the only factor that had a negative impact on the yield of the rLST (E-value = −1.137). However, it was not statistically significant (*p* > 0.05). Regarding temperature, induction time, and agitation speed, there were very slight non-statistically significant positive effects (*p* > 0.05) for shifting to the high level of these three variables (37 °C, 18 h, and 250 rpm, respectively) on the rLST yield (E-value = 1.101, 1.500, and 1.636, respectively). The rLST yield varied from 1.2 ± 0.57 mg/L to 50.5 ± 0.99 mg/L culture (Table S1).

**Figure 2:**
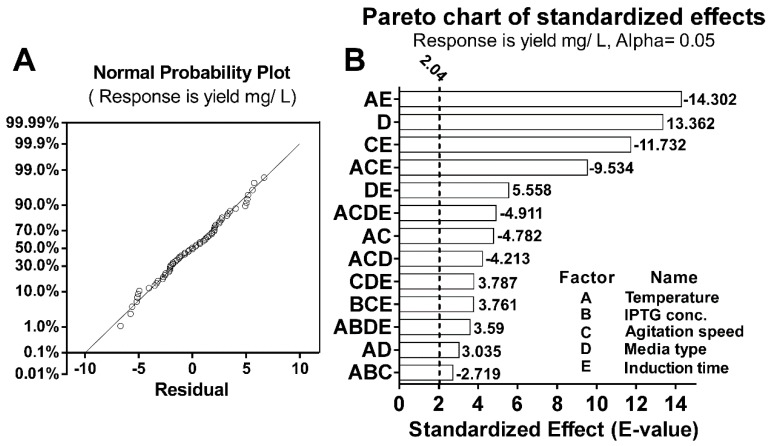
**Normal probability plot of residuals and Pareto chart of standardized effects.** (**A**). A normal probability plot of residuals. The straight line is a graphical representation of the mathematical regression equation and it represents the expected data, while the dots in the plots represents the actual observed data. (**B**). Pareto chart of standardized effects. Bars represent the significant interactions in the current model from the highest to the lowest regardless of the effect sign. The vertical dotted line represents the reference line and any factor that extends past this line is of significant effect at α = 0.05 (significance level). The magnitude of the E-value of the tested factor indicated its significance in affecting the response, while the positive or negative sign of the E-value was an indication of its positive or negative influence on the responses, respectively. In both charts, data was generated using Minitab 16 and plotted using GraphPad Prism (v6).

The strength of the model was tested by the normal probability plot of residuals (Figure 2A). The straight line in the graph represented the expected data, while the dots represented the actual observed data. A coefficient of (R = 0.95) suggested the fitted model could explain 95% of the total variation. The standardized effects’ magnitude and significance of each factor on its own or in combination with other factors are represented in the form of a Pareto chart (Figure 2B). Thirteen factors or factors’ interactions showed significant effects on the yield of the rLST, as indicated by the extension of their standardized effect beyond the reference line. Among those 13 factors/factors interactions, the growth media factor was the only standardized factor by itself that had a significant positive effect (E = 13.362). Moreover, it was the one with the highest positive significant effect. On the other hand, seven factor interactions had a significant negative effect with the interaction between the temperature and the induction time having the highest significant negative effect (E= −14.302). In addition, the interaction between the temperature, IPTG concentration, and the agitation speed had the least significant negative effect (E= −2.719). Lastly, the interaction between the temperature and the growth media had the lowest positive significant effect (E = 3.035).

**Figure 3:**
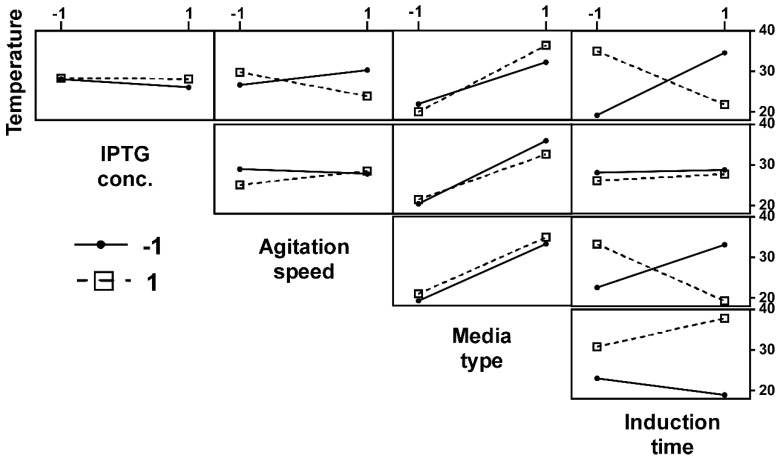
Interaction plot for the effects on rLST yield response. The interaction plot shows the possible interaction between the mean responses of factors under evaluation. The dotted line represents the high level (1) of the tested factor, while the solid line represents its low level (−1). The two levels (1 and −1) of the tested factors are: temperatures of 37 and 16 °C, IPTG concentration of 1 and 0.25 mM, Agitation speed of 250 and 100 rpm, media type of TSB and NB, and induction time of 18 and 3 h, respectively. Data was generated using Minitab 16 (Minitab, Inc., State College, Pennsylvania, PE, USA) and plotted using GraphPad Prism (v6) (GraphPad, California, CA, USA).

The interactions between different factors and their effect on the response are presented in the interaction plots (Figure 3). There was a strong interaction between the temperature and three other factors (induction time, agitation speed, and media type) even though the significance of the interactions varied. Meanwhile, it has a minimal interaction with IPTG concentration, which was not significant. In addition, induction time shows a strong interaction with both agitation speed and media type. However, there was a minimal interaction between the IPTG concentration and every other factor as well as the relationship between the agitation speed and the media type.

Taking all this into consideration, the optimum rLST yield was obtained in the 14th and 29th runs, both at rich media and high agitation speed levels, but with IPTG at a low level. High temperature level and low induction time were used in Run #14 and low temperature level and high induction time were used in Run #29.

### 3.3. Laboratory-Scale Production Cost of rLST

In order to evaluate the economic value of our applied system, we calculated the costs of rLST production using the conditions of Run #14 (taking into account the prices of: culture media, Ni-NTA beads, IPTG, and the size exclusion chromatography columns for buffer exchange, being the major contributors to the rLST production in our settings, in addition to the labor costs). Producing ~ 2666 units would cost about 7 EUR. Accordingly, the cost of one unit is ~0.003 EUR. Nevertheless, high scale production using our system will require further evaluation and more accurate calculations.

### 3.4. The rLST Is Active in Lysing Staphylococcal Cells

**Figure 4:**
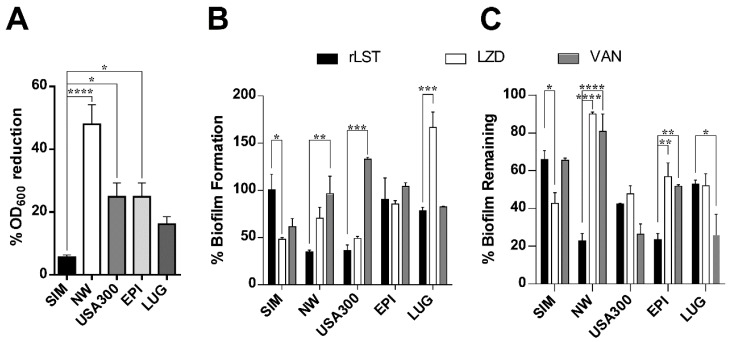
A. The rLST is active in-vitro against staphylococcal cells. (**A**). **Bacteriolytic activity assay**. Staphylococcal cells (*S. simulans*, SIM, *S. aureus* strain Newman, NW, *S. aureus* strain USA300, USA300, *S. epidermidis*, EPI, and *S. S. lugdunensis*, LUG) were suspended in 0.1 M phosphate buffer (pH 7.5) to final OD_600_ of 0.25, and then incubated with 50 µL of rLST at 37 °C. Data presented as the percent reduction in OD_600_ after 30 min. Bars represent the mean of four experiments and error bars are the standard error. Statistical analysis was done using a one-way ANOVA test followed by Dunnett’s multiple comparisons test (a *p* value of ≤0.05 was considered significant). (**B**). **Biofilm inhibition assay (Pre-exposure assay)**. Biofilm combating ability of rLST, black bars, linezolid (LZD), white bars, and vancomycin (VAN). Grey bars were tested using a crystal violet-based assay against the five staphylococcal cells mentioned above. Cells were grown in the absence and presence of the respective antimicrobial agent and, then, attached cells were stained with crystal violet. The fixed stain was then extracted with 99% ethanol and quantified spectrophotometrically at 595 nm. Bars represent the increases or decreases in biofilm formation compared to the biofilm formation level in the absence of the antimicrobial agents for each strain. Data presented is the average of two independent experiments with each one done in triplicate and error bars represent the standard error. (**C**). **Biofilm detachment assay (Post-exposure assay)**. Biofilms already formed by the five staphylococcal strains were exposed to 100X MIC of both LZD and VAN and 10 U rLST for 24 h at 37 °C. The remaining biofilms were quantified as described above. Data presented is the average of two independent experiments with each one done in triplicate and error bars represent the standard error. In both B and C, statistical analysis was done using two-way ANOVA, which was followed by Tukey’s multiple comparisons test with a significance level at *p* ≤ 0.05. In all three charts, * means *p* ≤ 0.05, ** means *p* ≤ 0.01, *** means *p* ≤ 0.001, and **** means *p* ≤ 0.0001. The charts were generated using GraphPad Prism (v6).

The rLST was tested against five staphylococcal strains in terms of reducing the OD_600_ of a cell suspension. As expected, rLST was totally non-effective against the immune species *S. simulans* strain (Figure 4A). The rLST showed the highest activity against the *S. aureus* strains especially strain Newman and was also effective against the MRSA strain (USA300). The reduction of the OD_600_ in both strains was statistically significant when compared to the immune cells of *S. simulans*. In addition, rLST showed significant activity against the commensal staphylococcal cells of *S. epidermidis* (Figure 4A). On the other hand, the activity of the rLST on the emerging in-dwelling device-related pathogen *S. lugdunensis* was very moderate and non-statistically significant from that on *S. simulans* (Figure 4A).

### 3.5. The rLST Is Active in Inhibiting and Combating Staphylococcal Biofilms

The clearest effect of rLST was shown in inhibiting the biofilm formation of the two *S. aureus* strains (Newman and USA300) when compared to vancomycin (Figure 4B). On the other hand, it did not show significant inhibition of the biofilm formation in either *S. epidermidis* nor *S. lugdunensis*. It was observed that *S. lugdunensis* in the presence of sub-MIC of linezolid showed enhanced biofilm formation (Figure 4B).

Upon testing the activity of the three antimicrobial agents in the detachment of already formed biofilms, *S. simulans* was the most resistant species to rLST while *S. aureus* Newman and *S. epidermidis* were the most sensitive (Figure 4C). The rLST showed significant superior activity over both linezolid and vancomycin in the detachment of the biofilms formed by the *S. aureus* strain Newman and *S. epidermidis*. However, in the case of strain USA300, both linezolid and vancomycin were capable of detaching already formed biofilms to a degree very comparable to that of rLST (Figure 4C).

### 3.6. The rLST Is Successfully Incorporated into a Nano-Emulgel and Released While Retaining Its Activity

**Figure 5:**
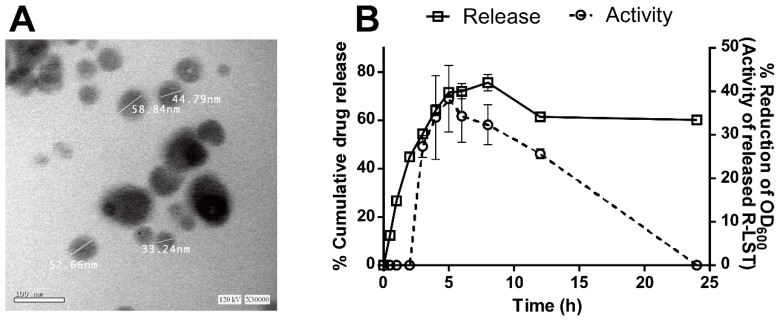
The recombinant lysostaphin (rLST) is successfully incorporated into a nano-emulgel and released while retaining its activity. (**A**) The formula particle is within the nano-range. High resolution-transmission electron microscopy image showing the globular structure of the prepared formula and displaying the size of the four representative nanoparticles. The photograph was obtained by an HR-TEM - JEOL2100 coupled with a Gatan axis-mount 2kx2k digital camera. (**B**). rLST is released from the formula in an active form. A chart showing the percentage of cumulative rLST release from the nano-emulgel formula (the left Y-axis and the solid line with open squares) over time in hours at the indicated time points (the x-axis). The released rLST was assayed for its bacteriolytic activity as measured by the decrease in the OD_600_ of the staphylococcal cells (the right Y-axis and the dotted line with open circles). The data presented is the average of two independent experiments each done in duplicate and the error bars represent the standard error. The charts were generated using GraphPad Prism (v6).

The prepared nano-emulgel was transparent with a yellowish color appearance. TEM examination showed spherical water globules with particle size-values ranging in the nano-size (Figure 5A). It was observed that rLST was released gradually over a prolonged time (Figure 5B) with the highest amount released after 8 h. The released fractions were analyzed using the activity assay and rLST showed the highest activity in the fraction released after 5 h. However, up to 8 h, the activity remained relatively high (Figure 5B).

### 3.7. The rLST Is Active in Curing S. aureus Skin Infection in Mice

**Figure 6:**
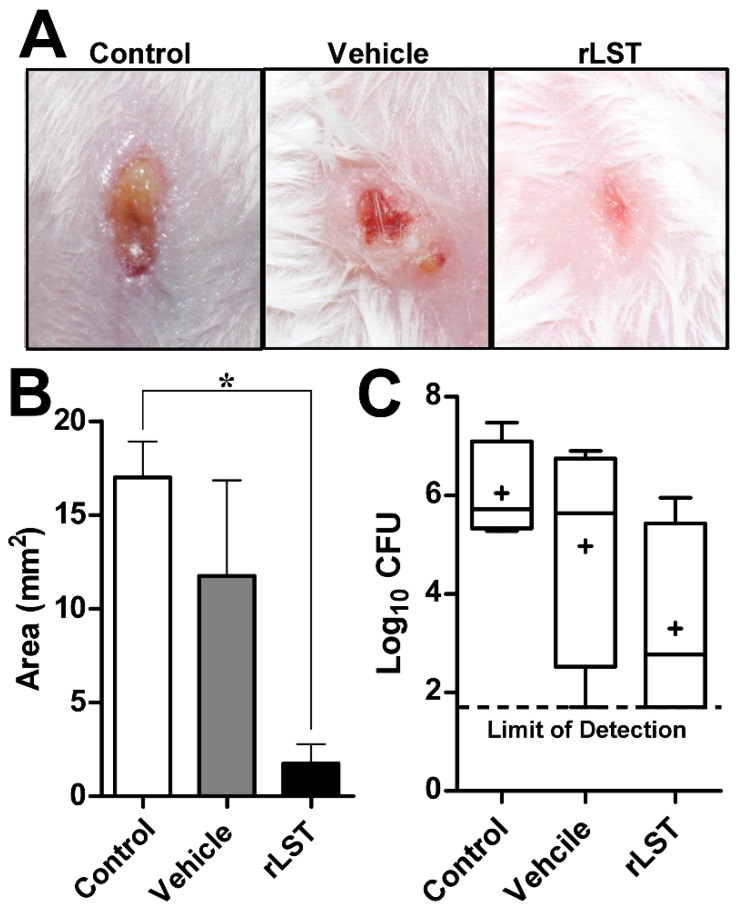
**The rLST is active in-vivo curing *S. aureus* skin infection in mice.** Mice were infected intradermally with approximately 3 × 10^7^ CFUs of *S. aureus* USA300. Forty-eight hours after infection, an open wound/abscess was formed at the site of injection. Mice were then treated topically, three times daily for five days, with either rLST or vehicle alone (Vehicle) and no treatment at all (Control). (**A**). Photographs of representative mice showing the infected and treated skin areas in the three groups. (**B**). Bar chart showing the lesion surface area (mm^2^) for the control group (white bar), vehicle group (grey bar), and R-LST (black bar). Statistical analysis was done using One-way ANOVA followed by Dunnett’s multiple comparisons test (a *p* * value of ≤0.05 was considered significant) and error bars represent the standard error. * indicates that the *p* value is ≤0.05. (**C**). Box plot of the bacterial burden recovered from the lesions of the mice of the three groups. The whiskers span the difference between the minimum and maximum readings, the horizontal bar represents the median, and the (+) sign represents the mean of the log_10_ CFU. The horizontal dotted line represents the limit of detection of the experiment, which is 1.69897 (log_10_ of 50 CFU). Statistical analysis was done using One-way ANOVA, which is followed by Dunnett’s multiple comparisons test (a *p* value of ≤0.05 was considered significant) and error bars represent the standard error.

Mice in the control group showed lesions that are ulcerated and filled with pus (Figure 6A) and the lesion extended over a large area. A similar trend was seen with the group treated with the vehicle only. On the other hand, in the mice treated with rLST, the lesions healed to a great extent and the size was significantly less than that of the control group (Figure 6B). The bacterial counts of *S. aureus* cells recovered from the mice group treated with the rLST yielded average counts that were 2.747 logs lower than those yielded from the control group and 1.671 logs lower than that of the vehicle-treated group (Figure 6C).

## 4. Discussion

In this study, we aimed to introduce a non-conventional antimicrobial agent to add to the antimicrobial arsenal needed to fight MDR-infections caused by *S. aureus*. Such an agent is urgently needed especially for countries with limited resources that cannot afford the costs of newly innovated antimicrobial agents. To this end, we optimized the production of recombinant lysostaphin expressed with the *E. coli* BL21 (DE3)/pET15b system to minimize both costs and time for production. Although this is not the first attempt to produce this biological macromolecule, we managed using factorial optimization to obtain a protein yield that surpassed comparable reported systems. For instance, previous studies used the *E. coli* BL21 (DE3) host combined with various expression systems including the pET28a yielded 22 mg/L [33], pET23b yielded 20 mg/L [34], pET15b yielded 11 mg/L [23], pET32a yielded 30 mg/L [35], and the current study yielded 50 mg/L.

Factorial design is a widely applied precise experimental tool that successfully studies the main factors effect and interaction effect of factors on the response. In the present study, five-factor two-level full factorial optimization experiments identified conditions that yielded up to 50 mg/L purified protein using as low as 0.25 mM IPTG. For the media type, the nutritious and buffered nature of TSB added an edge over the other two studied media and was the most significant factor in the main effects. Additionally, combining this with growth at 37 °C and good aeration of the culture (high agitation speed) managed to minimize the induction time to 3 h. It was also found that proper mixing via a high agitation rate is important for better induction.

For the purification purpose, we chose the Ni-NTA affinity chromatography since it allows simple one-step protein purification and does not require expertise or the equipment necessary for traditional protein purification schemes [35]. The relative high-cost of the nickel beads is overcome by the fact that they can be reused after being easily regenerated and recharged.

Overall, the production of pure active rLST took ~five hours while taking into consideration the induction time, sonication, purification, and preparatory steps. Cost-wise, the produced rLST costs ~ 0.003 EUR per unit of enzyme activity. This cost is very comparable to the cost reported by Szweda and co-workers [36]. However, the latter product was on a larger scale than that adopted in our current study. Accordingly, upscaling our production is expected to result in more savings per unit cost. Commercially, LST is available with around 137 EUR for 500 units, making the cost of one rLST unit 0.27 EUR. Due to the relatively high cost of this in-market product, commercial LST is not intended for clinical use.

Staphylococci are responsible for a significant proportion of the biofilm-based infections, which are challenging to healthcare facilities [37,38]. Biofilm combating ability of rLST using pre-exposure assay showed a superior biofilm inhibition effect on both the Newman strain and the MRSA strain USA300, (Figure 4B). As noted, sub-MIC concentrations of vancomycin and linezolid not only had a lower biofilm inhibition effect but also augmented the biofilm formation ability at certain instances such as in the case *S. lugdunensis* with linezolid, and USA300 when treated with vancomycin. The enhanced biofilm formation ability was previously explained as a response phenomenon to cell stress caused by cell wall-active antibiotics such as oxacillin, cephalothin, cephalexin, and vancomycin [39,40] and the protein synthesis inhibitor linezolid [41]. On the other hand, LST acts on the sessile staphylococci, destabilizing the whole biofilm matrix, but those staphylococci have to be LST-sensitive [28], which explains the strain-dependent pattern noticeable with rLST assays on preformed biofilms. Accordingly, rLST managed to significantly interfere with the formation of new biofilms and the maintenance of already formed ones.

The greatest fear that accompanies the introduction of an antimicrobial agent to the therapeutic field is the rapid development of resistance. Strandén and co-workers have reported the emergence of resistance to LST [42]. Yet, there are no reported LST-resistant mutants from in-vivo studies employing high doses of LST [10]. The most common resistance mechanism known for LST is the development of mutations in either the *femA* or the *femB* genes, which results in mono-glycine or tri-glycine cross bridges in the cell wall [42]. These resistant mutants were less fit and less virulent. Hence, the LST resistance caveat was easily overcome by adjunct beta-lactam antibiotics therapy [43].

The use of LST to treat infections has been demonstrated before, using different routes of administration, and it showed encouraging results modeling this bacteriocin as a promising antibiotics-alternative and a supporting adjuvant as well [44,45]. One of the most common LST administration routes was intravenous for staphylococcal infections of various degrees [44,45,46,47]. This route would be sensible for systemic infections. Yet, for skin infections, drug delivery through the skin is preferred for offering drug targeting ability over a prolonged period of time, and practicality of patient’s self-management of drug administration [48]. Moreover, one of the most appealing lysostaphin merits that makes it a suitable nominee for topical preparations above other formulas is its high specificity for staphylococcal species [49]. Therefore, it is unlikely to distress or negatively affect the normal microflora of the skin, which itself provides a protective barrier against colonizing or opportunistic pathogens [50].

Topical LST preparations previously reported included the use of petroleum-based creams [28], gel incorporated LST [50], and lysostaphin formulated in petroleum jelly [51]. These topical agents are normally very sticky with a lower spreading coefficient. Hence, they need to be applied with rubbing, which causes uneasiness of application and lowers patients’ adherence to treatment. Additionally, they tend to exhibit low stability [13]. Whereas, NEs are resistant to creaming, flocculation, and sedimentation of high stability. Another formula was made of chitosan-o/w cream incorporated with lysostaphin [52] that was useful for resolving *S. aureus* nasal colonization. Nasal application required a thick-based formula to stand in the face of mucociliary clearance that greatly limited LST delivery in the nasal cavity. However, topical skin preparations do not face such a problem and require other characteristics to make it more convenient to the patients. Moreover, the chitosan-o/w cream preparation procedure used seven components with heating and cooling of the aqueous and lipid phase before LST addition, which makes it more time and cost-consuming if considered for scaling-up.

Lysostaphin, which is a heat-labile protein, was required to be incorporated in a formula with a low-energy technique. The phase inversion composition (PIC) method, which required no heating, fulfilled this aim, with the edge of having an easy preparatory method. We were able to extend the LST release while maintaining its activity up to 8 h. This prolonged rLST release was expected since it was incorporated in the internal phase of the nano-emulgel. Therefore, the drug should be diffused from the internal phase to the external phase and then to the release media in order to be detected. To test the efficacy of the formula, the murine skin model was chosen since abscesses are the hallmarks of staphylococcal infections. Abscess lesions tend to harbor high titers of slowly multiplying bacteria, which makes antibiotic failure highly likely to happen [10]. The rLST managed to enhance the recovery of the mice from the infection, as indicated by the decrease of both the surface area of the formed lesions and the bacterial burden within the skin.

## 5. Conclusions

The current study models an expeditious rLST production protocol from a simple rapid enzyme expression directly to a topical formula of high commercial potential as an alternative antimicrobial agent for treating methicillin-resistant *S. aureus* skin infections. This work establishes a rLST production platform that aims to satisfy the needs of countries with limited resources in order to be able to combat the surge in the MDR *S. aureus* infections.

## Supplementary Materials

The following are available online at https://www.mdpi.com/2218-273X/10/3/435/s1.
