# Supplementary material for: A Rapid Lysostaphin Production Approach and a Convenient Novel Lysostaphin Loaded Nano-emulgel; As a Sustainable Low-Cost Methicillin-Resistant Staphylococcus aureus Combating Platform
Source: Biomolecules. 2020 Mar 12;10(3):435. doi: 10.3390/biom10030435 (PMC7175171; doi:10.3390/biom10030435)
Supplement: Supplementary file 1 [file biomolecules-10-00435-s001.pdf]

Supplementary material

Table 1. Full factorial matrix representing the coded values for independent factors for recombinant lysostaphin production.

Run	Factors					Response Mean ± SD
	Temperature	IPTG concentration	Agitation speed	Media type	Induction time	
**1**	-1	-1	-1	-1	-1	13 ± 0.99
**2**	1	-1	-1	-1	-1	16.65 ± 1.77
**3**	-1	1	-1	-1	-1	6.1 ± 0.42
**4**	1	1	-1	-1	-1	21.9 ± 2.55
**5**	-1	-1	1	-1	-1	22 ± 2.83
**6**	1	-1	1	-1	-1	41.1 ± 8.20
**7**	-1	1	1	-1	-1	23.4 ± 2.83
**8**	1	1	1	-1	-1	39.95 ± 3.61
**9**	-1	-1	-1	1	-1	18.8 ± 3.82
**10**	1	-1	-1	1	-1	32.7 ± 7.92
**11**	-1	1	-1	1	-1	21.65 ± 2.05
**12**	1	1	-1	1	-1	31 ± 7.07
**13**	-1	-1	1	1	-1	27 ± 1.41
**14**	1	-1	1	1	-1	50.5 ± 0.99
**15**	-1	1	1	1	-1	21.65 ± 2.62
**16**	1	1	1	1	-1	43.15 ± 4.74
**17**	-1	-1	-1	-1	1	32.75 ± 2.90
**18**	1	-1	-1	-1	1	20.25 ± 2.19
**19**	-1	1	-1	-1	1	31.4 ± 4.95
**20**	1	1	-1	-1	1	19 ± 4.53
**21**	-1	-1	1	-1	1	16.55 ± 3.04
**22**	1	-1	1	-1	1	1.2 ± 0.57
**23**	-1	1	1	-1	1	30.35 ± 7.00
**24**	1	1	1	-1	1	-
**25**	-1	-1	-1	1	1	45.1 ± 7.21
**26**	1	-1	-1	1	1	49.1 ± 3.54
**27**	-1	1	-1	1	1	22.3 ± 2.97
**28**	1	1	-1	1	1	47.7 ± 7.35
**29**	-1	-1	1	1	1	49.8 ± 3.96
**30**	1	-1	1	1	1	15.05 ± 5.73
**31**	-1	1	1	1	1	51.8 ± 9.48
**32**	1	1	1	1	1	22.15 ± 1.63
**33**	0	0	0	0	0	8.45 ± 3.18

*"0" represents the original value of each component under the control conditions, "-" represents the low level and "+" represents the high level for each factor.
